# (*E*)-*N*′-[3-(4-Chloro­benzo­yloxy)benzyl­idene]pyridine-4-carbohydrazide acetic acid monosolvate monohydrate

**DOI:** 10.1107/S1600536812017369

**Published:** 2012-04-25

**Authors:** Chun-Hua Diao, Zhi Fan

**Affiliations:** a College of Sciences, Tianjin University of Science and Technology, Tianjin 300457, People’s Republic of China

## Abstract

In the Schiff base mol­ecule of the title compound, C_20_H_14_ClN_3_O_3_·CH_3_COOH·H_2_O, the central benzene ring makes dihedral angles of 36.26 (7) and 27.59 (8)°, respectively, with the terminal chloro­phenyl and pyridine rings. In the crystal, the three components are linked by O—H⋯O, N—H⋯O, O—H⋯N and C—H⋯O hydrogen bonds into a double-tape structure along the *a* axis.

## Related literature

For general background to the use of Schiff base derivatives in the development of protein and enzyme mimics, see: Santos *et al.* (2001 ▶). For closely related crystal structures, see: Diao *et al.* (2007 ▶); Peralta *et al.* (2007 ▶); de Souza *et al.* (2007 ▶); Wardell *et al.* (2005 ▶). For reference bond-length data, see: Allen *et al.* (1987 ▶).
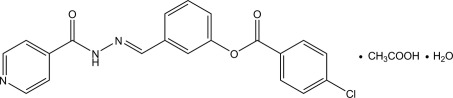


## Experimental

### 

#### Crystal data

C_20_H_14_ClN_3_O_3_·C_2_H_4_O_2_·H_2_O
*M* *_r_* = 457.86
Triclinic, 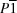

*a* = 6.6666 (15) Å
*b* = 7.5437 (17) Å
*c* = 24.781 (6) Å
α = 81.526 (4)°
β = 82.969 (4)°
γ = 66.632 (4)°
*V* = 1128.7 (5) Å^3^
*Z* = 2
Mo *K*α radiation
μ = 0.21 mm^−1^
*T* = 294 K
0.18 × 0.16 × 0.10 mm

#### Data collection

Bruker SMART APEX CCD area-detector diffractometer
Absorption correction: multi-scan (*SADABS*; Sheldrick, 1996 ▶) *T*_min_ = 0.928, *T*_max_ = 0.979
5755 measured reflections
3936 independent reflections
2501 reflections with *I* > 2σ(*I*)
*R*_int_ = 0.019

#### Refinement

*R*[*F* ^2^ > 2σ(*F* ^2^)] = 0.043
*wR*(*F* ^2^) = 0.119
*S* = 1.03
3936 reflections
298 parameters
3 restraints
H atoms treated by a mixture of independent and constrained refinement
Δρ_max_ = 0.15 e Å^−3^
Δρ_min_ = −0.17 e Å^−3^

### 

Data collection: *SMART* (Bruker, 1999 ▶); cell refinement: *SAINT* (Bruker, 1999 ▶); data reduction: *SAINT*; program(s) used to solve structure: *SHELXS97* (Sheldrick, 2008 ▶); program(s) used to refine structure: *SHELXL97* (Sheldrick, 2008 ▶); molecular graphics: *SHELXTL* (Sheldrick, 2008 ▶); software used to prepare material for publication: *SHELXTL*.

## Supplementary Material

Crystal structure: contains datablock(s) global. DOI: 10.1107/S1600536812017369/is5118sup1.cif

Additional supplementary materials:  crystallographic information; 3D view; checkCIF report

## Figures and Tables

**Table 1:** Hydrogen-bond geometry (Å, °)

*D*—H⋯*A*	*D*—H	H⋯*A*	*D*⋯*A*	*D*—H⋯*A*
O6—H6*A*⋯O3	0.86 (3)	2.00 (3)	2.843 (2)	166 (2)
O6—H6*B*⋯O4^i^	0.86 (2)	1.98 (2)	2.812 (3)	165 (3)
O5—H5*A*⋯N3^ii^	0.82	1.85	2.646 (3)	164
N2—H2⋯O6^iii^	0.86	2.04	2.879 (2)	164
C14—H14⋯O6^iii^	0.93	2.59	3.367 (4)	141
C17—H17⋯O6^iii^	0.93	2.50	3.331 (3)	149

Symmetry codes: (i) 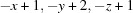; (ii) 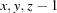; (iii) 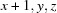.

**Fig. 1.**
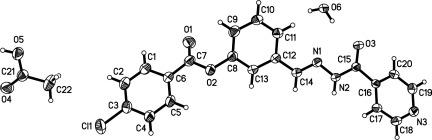
The structure of the title compound with displacement ellipsoids for non-H atoms drawn at the 50% probability level.

**Fig. 2.**
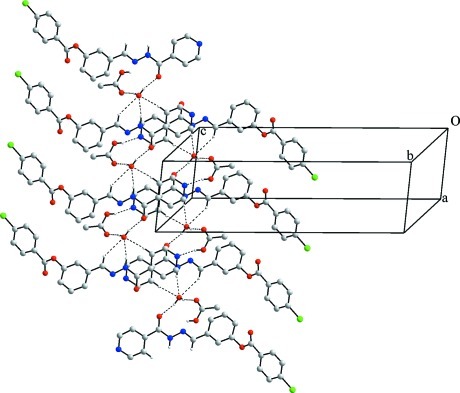
A packing diagram for the title compound with the hydrogen bonds drawn as dashed lines. H atoms not involved in the hydrogen bonds have been omitted.

## supplementary crystallographic information

### Comment

Many Schiff base derivatives have been synthesized and found to exhibit important pharmacological properties, such as antibacterial, antitumor and antitoxic activities (Santos *et al.*, 2001). Among the large number of the compounds, isonicotinohydrazide forms a variety of Schiff bases with aldehydes, and the synthesis and crystal structures of some of them have been reported (Wardell *et al.*, 2005; Peralta *et al.*, 2007; de Souza *et al.*, 2007).

In order to obtain more detailed information on the structural conformation of the molecule that may be of value in structure-activity analysis, we report here the synthesis and structure of the title compound, (I), as part of our study of isonicotinoylhydrazones (Diao *et al.*, 2007).

In (I) (Fig. 1), the central benzene ring (C8–C14/O2) is nearly planar, with an r.m.s. deviation for fitted atoms of 0.0255 Å. This plane makes dihedral angles of 27.59 (8) and 36.26 (7)° with the pyridine ring (C16–C20/N3) and the terminal benzene ring (C1–C6),respectively. The dihedral angle between the pyridine ring and the benzene ring is 26.03 (7)°. All bond lengths are within normal ranges (Allen *et al.*, 1987).

The acetic acid molecule and the water molecule are effectively tethered to the Schiff base component by a combination of independent hydrogen bonds, two O—H···O, one N—H···O, one O—H···N and one C—H···O types (Fig. 2 and Table 2).

### Experimental

An anhydrous ethanol solution (50 ml) of 3-formylphenyl 4-chlorobenzoate (2.61 g, 10 mmol) was added to an anhydrous ethanol solution (50 ml) of isonicotinohydrazide (1.37 g, 10 mmol) and the mixture stirred at 350 K for 5 h under N_2_, giving a white precipitate. The product was isolated, recrystallized from ethanol, and then dried in a vacuum to give pure compound (I) in 72% yield. Colorless single crystals of (I) suitable for X-ray analysis were obtained by slow evaporation of a solution of ethanol and acetic acid (80:20 *v*/*v*).

### Refinement

The H atoms of the water molecules were located in a difference Fourier map and refined, with distance restraints of O—H = 0.85 (1) and H···H = 1.45 (1) Å, and with *U*_iso_(H) = 1.5*U*_eq_(O). Other H atoms were included in calculated positions and refined using a riding model approximation. Constrained C—H and N—H bond lengths and *U*_iso_(H) values: 0.93 Å and 1.2*U*_eq_(C) for C*sp*^2^—H; 0.97 Å and 1.2*U*_eq_(C) for methylene C—H; 0.82 Å and 1.5*U*_eq_(O) for hydroxyl O—H; 0.86 Å and 1.2*U*_eq_(N) for imino N—H.

### Crystal data

**Table:**

C_20_H_14_ClN_3_O_3_·C_2_H_4_O_2_·H_2_O	*Z* = 2
*M**_r_* = 457.86	*F*(000) = 476
Triclinic, *P*1	*D*_x_ = 1.347 Mg m^−^^3^
Hall symbol: -P 1	Mo *K*α radiation, λ = 0.71073 Å
*a* = 6.6666 (15) Å	Cell parameters from 1984 reflections
*b* = 7.5437 (17) Å	θ = 2.5–25.2°
*c* = 24.781 (6) Å	µ = 0.21 mm^−^^1^
α = 81.526 (4)°	*T* = 294 K
β = 82.969 (4)°	Block, colorless
γ = 66.632 (4)°	0.18 × 0.16 × 0.10 mm
*V* = 1128.7 (5) Å^3^	

### Data collection

**Table:**

Bruker SMART APEX CCD area-detector diffractometer	3936 independent reflections
Radiation source: fine-focus sealed tube	2501 reflections with *I* > 2σ(*I*)
Graphite monochromator	*R*_int_ = 0.019
φ and ω scans	θ_max_ = 25.0°, θ_min_ = 1.7°
Absorption correction: multi-scan (*SADABS*; Sheldrick, 1996)	*h* = −6→7
*T*_min_ = 0.928, *T*_max_ = 0.979	*k* = −8→8
5755 measured reflections	*l* = −27→29

### Refinement

**Table:**

Refinement on *F*^2^	Secondary atom site location: difference Fourier map
Least-squares matrix: full	Hydrogen site location: inferred from neighbouring sites
*R*[*F*^2^ > 2σ(*F*^2^)] = 0.043	H atoms treated by a mixture of independent and constrained refinement
*wR*(*F*^2^) = 0.119	*w* = 1/[σ^2^(*F*_o_^2^) + (0.0473*P*)^2^ + 0.2041*P*] where *P* = (*F*_o_^2^ + 2*F*_c_^2^)/3
*S* = 1.03	(Δ/σ)_max_ = 0.001
3936 reflections	Δρ_max_ = 0.15 e Å^−^^3^
298 parameters	Δρ_min_ = −0.17 e Å^−^^3^
3 restraints	Extinction correction: *SHELXL97* (Sheldrick, 2008), Fc^*^=kFc[1+0.001xFc^2^λ^3^/sin(2θ)]^-1/4^
Primary atom site location: structure-invariant direct methods	Extinction coefficient: 0.0070 (17)

### Special details

**Table:**

Geometry. All e.s.d.'s (except the e.s.d. in the dihedral angle between two l.s. planes) are estimated using the full covariance matrix. The cell e.s.d.'s are taken into account individually in the estimation of e.s.d.'s in distances, angles and torsion angles; correlations between e.s.d.'s in cell parameters are only used when they are defined by crystal symmetry. An approximate (isotropic) treatment of cell e.s.d.'s is used for estimating e.s.d.'s involving l.s. planes.
Refinement. Refinement of *F*^2^ against ALL reflections. The weighted *R*-factor *wR* and goodness of fit *S* are based on *F*^2^, conventional *R*-factors *R* are based on *F*, with *F* set to zero for negative *F*^2^. The threshold expression of *F*^2^ > 2σ(*F*^2^) is used only for calculating *R*-factors(gt) *etc*. and is not relevant to the choice of reflections for refinement. *R*-factors based on *F*^2^ are statistically about twice as large as those based on *F*, and *R*- factors based on ALL data will be even larger.

### Fractional atomic coordinates and isotropic or equivalent isotropic displacement parameters (Å^2^)

**Table:**

	*x*	*y*	*z*	*U*_iso_*/*U*_eq_	
Cl1	1.33537 (13)	0.76087 (12)	0.41389 (3)	0.0905 (3)	
O1	0.5002 (3)	0.8179 (3)	0.59922 (8)	0.0951 (7)	
O2	0.7889 (3)	0.6503 (3)	0.64925 (7)	0.0686 (5)	
O3	0.3270 (2)	0.7186 (2)	0.98573 (7)	0.0608 (5)	
N1	0.5560 (3)	0.6798 (3)	0.88899 (8)	0.0524 (5)	
N2	0.6335 (3)	0.7116 (3)	0.93397 (7)	0.0534 (5)	
H2	0.7581	0.7221	0.9319	0.064*	
N3	0.7533 (4)	0.8006 (3)	1.12485 (8)	0.0626 (6)	
C1	0.7913 (5)	0.7979 (5)	0.50434 (12)	0.0820 (9)	
H1	0.6443	0.8341	0.4987	0.098*	
C2	0.9364 (5)	0.8039 (4)	0.46046 (11)	0.0809 (9)	
H2A	0.8882	0.8445	0.4254	0.097*	
C3	1.1507 (4)	0.7501 (4)	0.46886 (10)	0.0628 (7)	
C4	1.2231 (5)	0.6916 (5)	0.52037 (11)	0.0822 (9)	
H4	1.3702	0.6559	0.5258	0.099*	
C5	1.0767 (4)	0.6859 (4)	0.56408 (11)	0.0742 (8)	
H5	1.1258	0.6454	0.5991	0.089*	
C6	0.8596 (4)	0.7392 (3)	0.55674 (10)	0.0563 (6)	
C7	0.6938 (4)	0.7422 (4)	0.60230 (11)	0.0643 (7)	
C8	0.6574 (4)	0.6336 (4)	0.69716 (10)	0.0573 (6)	
C9	0.4946 (4)	0.5648 (4)	0.69816 (11)	0.0669 (7)	
H9	0.4609	0.5348	0.6662	0.080*	
C10	0.3821 (4)	0.5412 (4)	0.74740 (11)	0.0659 (7)	
H10	0.2705	0.4958	0.7486	0.079*	
C11	0.4328 (4)	0.5838 (3)	0.79477 (10)	0.0580 (6)	
H11	0.3540	0.5691	0.8276	0.070*	
C12	0.6014 (4)	0.6488 (3)	0.79379 (9)	0.0511 (6)	
C13	0.7122 (4)	0.6747 (4)	0.74412 (10)	0.0573 (6)	
H13	0.8239	0.7201	0.7426	0.069*	
C14	0.6656 (4)	0.6871 (4)	0.84357 (10)	0.0581 (6)	
H14	0.7887	0.7173	0.8421	0.070*	
C15	0.5077 (4)	0.7258 (3)	0.98161 (9)	0.0474 (6)	
C16	0.6030 (3)	0.7529 (3)	1.03005 (9)	0.0450 (5)	
C17	0.8176 (4)	0.7278 (4)	1.03257 (10)	0.0626 (7)	
H17	0.9165	0.6951	1.0022	0.075*	
C18	0.8846 (4)	0.7516 (4)	1.08031 (11)	0.0713 (8)	
H18	1.0309	0.7321	1.0814	0.086*	
C19	0.5476 (4)	0.8251 (4)	1.12229 (10)	0.0636 (7)	
H19	0.4521	0.8590	1.1532	0.076*	
C20	0.4667 (4)	0.8032 (4)	1.07641 (10)	0.0594 (7)	
H20	0.3199	0.8225	1.0767	0.071*	
O4	1.0439 (3)	0.9861 (3)	0.19590 (9)	0.0936 (7)	
O5	0.8367 (4)	0.8213 (3)	0.22488 (7)	0.0878 (6)	
H5A	0.8297	0.8234	0.1920	0.132*	
C21	0.9595 (5)	0.9109 (4)	0.23256 (11)	0.0660 (7)	
C22	0.9809 (6)	0.9134 (5)	0.29172 (12)	0.1010 (11)	
H22A	1.1128	0.9314	0.2958	0.151*	
H22B	0.9867	0.7924	0.3116	0.151*	
H22C	0.8570	1.0181	0.3058	0.151*	
O6	0.0290 (3)	0.7824 (3)	0.90528 (8)	0.0777 (6)	
H6A	0.134 (4)	0.765 (4)	0.9249 (10)	0.117*	
H6B	0.018 (5)	0.864 (4)	0.8768 (8)	0.117*	

### Atomic displacement parameters (Å^2^)

**Table:**

	*U*^11^	*U*^22^	*U*^33^	*U*^12^	*U*^13^	*U*^23^
Cl1	0.0937 (6)	0.1030 (6)	0.0582 (5)	−0.0225 (5)	0.0095 (4)	−0.0146 (4)
O1	0.0563 (12)	0.1292 (18)	0.0847 (15)	−0.0278 (12)	−0.0173 (10)	0.0215 (12)
O2	0.0556 (10)	0.0987 (13)	0.0469 (10)	−0.0248 (10)	−0.0071 (8)	−0.0053 (9)
O3	0.0446 (9)	0.0839 (12)	0.0618 (11)	−0.0325 (9)	−0.0085 (8)	−0.0052 (9)
N1	0.0542 (12)	0.0602 (12)	0.0502 (12)	−0.0289 (10)	−0.0117 (10)	−0.0023 (9)
N2	0.0512 (11)	0.0751 (13)	0.0467 (12)	−0.0376 (10)	−0.0089 (9)	−0.0021 (9)
N3	0.0690 (14)	0.0746 (14)	0.0544 (13)	−0.0382 (12)	−0.0109 (11)	−0.0031 (10)
C1	0.0660 (18)	0.117 (3)	0.0624 (19)	−0.0361 (17)	−0.0226 (15)	0.0082 (16)
C2	0.083 (2)	0.104 (2)	0.0537 (18)	−0.0337 (18)	−0.0217 (16)	0.0064 (15)
C3	0.0722 (17)	0.0602 (15)	0.0519 (15)	−0.0195 (14)	−0.0023 (13)	−0.0129 (12)
C4	0.0588 (17)	0.123 (3)	0.0561 (18)	−0.0247 (17)	−0.0066 (14)	−0.0115 (16)
C5	0.0655 (17)	0.107 (2)	0.0449 (15)	−0.0256 (16)	−0.0122 (13)	−0.0066 (14)
C6	0.0581 (15)	0.0588 (15)	0.0527 (15)	−0.0211 (12)	−0.0122 (12)	−0.0056 (11)
C7	0.0613 (17)	0.0715 (17)	0.0618 (17)	−0.0257 (14)	−0.0178 (14)	−0.0013 (13)
C8	0.0514 (14)	0.0650 (16)	0.0524 (15)	−0.0184 (13)	−0.0058 (12)	−0.0071 (12)
C9	0.0721 (17)	0.0750 (18)	0.0613 (17)	−0.0312 (15)	−0.0091 (14)	−0.0189 (13)
C10	0.0687 (17)	0.0780 (18)	0.0680 (18)	−0.0430 (15)	−0.0068 (14)	−0.0153 (14)
C11	0.0581 (15)	0.0636 (16)	0.0588 (16)	−0.0295 (13)	−0.0036 (12)	−0.0091 (12)
C12	0.0498 (13)	0.0557 (14)	0.0495 (14)	−0.0215 (11)	−0.0086 (11)	−0.0039 (11)
C13	0.0500 (14)	0.0720 (17)	0.0562 (16)	−0.0297 (13)	−0.0100 (12)	−0.0034 (12)
C14	0.0566 (15)	0.0740 (17)	0.0550 (16)	−0.0373 (13)	−0.0093 (12)	−0.0025 (12)
C15	0.0464 (13)	0.0474 (13)	0.0507 (14)	−0.0217 (11)	−0.0097 (11)	0.0034 (10)
C16	0.0433 (12)	0.0474 (13)	0.0475 (13)	−0.0231 (10)	−0.0060 (10)	0.0033 (10)
C17	0.0501 (14)	0.0955 (19)	0.0517 (15)	−0.0379 (14)	−0.0007 (11)	−0.0106 (13)
C18	0.0582 (16)	0.108 (2)	0.0625 (18)	−0.0458 (16)	−0.0087 (14)	−0.0108 (16)
C19	0.0685 (17)	0.0755 (18)	0.0487 (15)	−0.0307 (15)	0.0001 (12)	−0.0076 (12)
C20	0.0481 (14)	0.0770 (17)	0.0574 (16)	−0.0305 (13)	−0.0008 (12)	−0.0045 (13)
O4	0.0878 (14)	0.1262 (18)	0.0788 (14)	−0.0620 (14)	−0.0170 (11)	0.0218 (12)
O5	0.1197 (17)	0.1169 (16)	0.0548 (12)	−0.0778 (15)	−0.0107 (12)	0.0026 (12)
C21	0.0703 (18)	0.0653 (17)	0.0599 (17)	−0.0235 (15)	−0.0166 (14)	0.0033 (13)
C22	0.146 (3)	0.096 (2)	0.068 (2)	−0.049 (2)	−0.040 (2)	0.0006 (17)
O6	0.0587 (11)	0.1268 (17)	0.0607 (12)	−0.0549 (12)	−0.0152 (9)	0.0147 (11)

### Geometric parameters (Å, º)

**Table:**

Cl1—C3	1.736 (3)	C10—C11	1.375 (3)
O1—C7	1.194 (3)	C10—H10	0.9300
O2—C7	1.353 (3)	C11—C12	1.389 (3)
O2—C8	1.408 (3)	C11—H11	0.9300
O3—C15	1.218 (2)	C12—C13	1.385 (3)
N1—C14	1.271 (3)	C12—C14	1.456 (3)
N1—N2	1.373 (2)	C13—H13	0.9300
N2—C15	1.353 (3)	C14—H14	0.9300
N2—H2	0.8600	C15—C16	1.499 (3)
N3—C19	1.317 (3)	C16—C20	1.374 (3)
N3—C18	1.322 (3)	C16—C17	1.375 (3)
C1—C2	1.372 (4)	C17—C18	1.374 (3)
C1—C6	1.380 (3)	C17—H17	0.9300
C1—H1	0.9300	C18—H18	0.9300
C2—C3	1.356 (4)	C19—C20	1.373 (3)
C2—H2A	0.9300	C19—H19	0.9300
C3—C4	1.368 (3)	C20—H20	0.9300
C4—C5	1.374 (4)	O4—C21	1.200 (3)
C4—H4	0.9300	O5—C21	1.295 (3)
C5—C6	1.368 (3)	O5—H5A	0.8200
C5—H5	0.9300	C21—C22	1.494 (4)
C6—C7	1.476 (4)	C22—H22A	0.9600
C8—C9	1.372 (3)	C22—H22B	0.9600
C8—C13	1.373 (3)	C22—H22C	0.9600
C9—C10	1.379 (3)	O6—H6A	0.854 (10)
C9—H9	0.9300	O6—H6B	0.852 (10)
			
C7—O2—C8	119.8 (2)	C13—C12—C11	118.7 (2)
C14—N1—N2	116.74 (19)	C13—C12—C14	119.7 (2)
C15—N2—N1	117.92 (19)	C11—C12—C14	121.6 (2)
C15—N2—H2	121.0	C8—C13—C12	120.2 (2)
N1—N2—H2	121.0	C8—C13—H13	119.9
C19—N3—C18	116.7 (2)	C12—C13—H13	119.9
C2—C1—C6	121.2 (3)	N1—C14—C12	121.0 (2)
C2—C1—H1	119.4	N1—C14—H14	119.5
C6—C1—H1	119.4	C12—C14—H14	119.5
C3—C2—C1	119.2 (3)	O3—C15—N2	123.0 (2)
C3—C2—H2A	120.4	O3—C15—C16	121.0 (2)
C1—C2—H2A	120.4	N2—C15—C16	116.0 (2)
C2—C3—C4	120.9 (3)	C20—C16—C17	116.8 (2)
C2—C3—Cl1	119.7 (2)	C20—C16—C15	117.7 (2)
C4—C3—Cl1	119.4 (2)	C17—C16—C15	125.5 (2)
C3—C4—C5	119.5 (3)	C18—C17—C16	119.4 (2)
C3—C4—H4	120.2	C18—C17—H17	120.3
C5—C4—H4	120.2	C16—C17—H17	120.3
C6—C5—C4	120.8 (2)	N3—C18—C17	123.8 (2)
C6—C5—H5	119.6	N3—C18—H18	118.1
C4—C5—H5	119.6	C17—C18—H18	118.1
C5—C6—C1	118.4 (3)	N3—C19—C20	123.4 (2)
C5—C6—C7	123.2 (2)	N3—C19—H19	118.3
C1—C6—C7	118.4 (2)	C20—C19—H19	118.3
O1—C7—O2	123.4 (3)	C19—C20—C16	119.9 (2)
O1—C7—C6	125.3 (2)	C19—C20—H20	120.0
O2—C7—C6	111.3 (2)	C16—C20—H20	120.0
C9—C8—C13	121.3 (2)	C21—O5—H5A	109.5
C9—C8—O2	122.2 (2)	O4—C21—O5	123.3 (3)
C13—C8—O2	116.3 (2)	O4—C21—C22	123.9 (3)
C8—C9—C10	118.7 (2)	O5—C21—C22	112.8 (3)
C8—C9—H9	120.6	C21—C22—H22A	109.5
C10—C9—H9	120.6	C21—C22—H22B	109.5
C11—C10—C9	120.8 (2)	H22A—C22—H22B	109.5
C11—C10—H10	119.6	C21—C22—H22C	109.5
C9—C10—H10	119.6	H22A—C22—H22C	109.5
C10—C11—C12	120.3 (2)	H22B—C22—H22C	109.5
C10—C11—H11	119.9	H6A—O6—H6B	115.3 (17)
C12—C11—H11	119.9		
			
C14—N1—N2—C15	172.9 (2)	C10—C11—C12—C13	1.8 (3)
C6—C1—C2—C3	0.4 (5)	C10—C11—C12—C14	−177.3 (2)
C1—C2—C3—C4	−0.5 (5)	C9—C8—C13—C12	−0.6 (4)
C1—C2—C3—Cl1	−178.9 (2)	O2—C8—C13—C12	−175.4 (2)
C2—C3—C4—C5	0.5 (5)	C11—C12—C13—C8	−1.0 (3)
Cl1—C3—C4—C5	178.9 (2)	C14—C12—C13—C8	178.0 (2)
C3—C4—C5—C6	−0.4 (5)	N2—N1—C14—C12	177.86 (19)
C4—C5—C6—C1	0.3 (4)	C13—C12—C14—N1	174.2 (2)
C4—C5—C6—C7	−177.9 (3)	C11—C12—C14—N1	−6.7 (4)
C2—C1—C6—C5	−0.3 (5)	N1—N2—C15—O3	−2.7 (3)
C2—C1—C6—C7	178.1 (3)	N1—N2—C15—C16	177.73 (18)
C8—O2—C7—O1	2.7 (4)	O3—C15—C16—C20	−11.2 (3)
C8—O2—C7—C6	−178.4 (2)	N2—C15—C16—C20	168.4 (2)
C5—C6—C7—O1	166.5 (3)	O3—C15—C16—C17	167.4 (2)
C1—C6—C7—O1	−11.8 (4)	N2—C15—C16—C17	−13.0 (3)
C5—C6—C7—O2	−12.4 (4)	C20—C16—C17—C18	0.7 (4)
C1—C6—C7—O2	169.4 (2)	C15—C16—C17—C18	−178.0 (2)
C7—O2—C8—C9	50.5 (3)	C19—N3—C18—C17	0.7 (4)
C7—O2—C8—C13	−134.6 (2)	C16—C17—C18—N3	−1.0 (4)
C13—C8—C9—C10	1.4 (4)	C18—N3—C19—C20	−0.3 (4)
O2—C8—C9—C10	176.0 (2)	N3—C19—C20—C16	0.1 (4)
C8—C9—C10—C11	−0.6 (4)	C17—C16—C20—C19	−0.3 (3)
C9—C10—C11—C12	−1.0 (4)	C15—C16—C20—C19	178.5 (2)

### Hydrogen-bond geometry (Å, º)

**Table:**

*D*—H···*A*	*D*—H	H···*A*	*D*···*A*	*D*—H···*A*
O6—H6*A*···O3	0.86 (3)	2.00 (3)	2.843 (2)	166 (2)
O6—H6*B*···O4^i^	0.86 (2)	1.98 (2)	2.812 (3)	165 (3)
O5—H5*A*···N3^ii^	0.82	1.85	2.646 (3)	164
N2—H2···O6^iii^	0.86	2.04	2.879 (2)	164
C14—H14···O6^iii^	0.93	2.59	3.367 (4)	141
C17—H17···O6^iii^	0.93	2.50	3.331 (3)	149

Symmetry codes: (i) −*x*+1, −*y*+2, −*z*+1; (ii) *x*, *y*, *z*−1; (iii) *x*+1, *y*, *z*.
